# Design and manufacture of one-size-fits-all healthcare face shields for the NHS during the COVID-19 pandemic

**DOI:** 10.1016/j.heliyon.2023.e19368

**Published:** 2023-09-04

**Authors:** Urvashi F. Gunputh, Gavin Williams, Adam Leighton, Wayne Carter, Hirbod Varasteh, Marzena Pawlik, Yiling Lu, Ruth Sims, Melinda Lyons, Jenny Clementson, Gyan Tripathi, Nick Chambers, Matt Roe, Paul Wood

**Affiliations:** a Institute for Innovation in Sustainable Engineering, IISE, University of Derby, UK; b School of Psychology, University of Derby, UK; c School of Human Sciences, University of Derby, UK; d University Hospitals of Derby and Burton NHS Foundation Trust, Derby, UK; e Riverside Medical Packaging Company Ltd., Derby, UK

**Keywords:** COVID-19, ppe, Face Shield, One-size-fits-all, Coefficient of friction, Ergonomics

## Abstract

During the COVID-19 pandemic, there was a shortage of personal protective equipment, PPE, which resulted in non-certified PPE being used by healthcare staffs. These would not provide the appropriate protection against the SARS-CoV-2 virus. Together with the local NHS Trust (University Hospitals of Derby and Burton (UHDB) NHS Foundation Trust) and a local small and medium enterprise (SME), Riverside Medical Packaging Ltd, the University of Derby (UoD) developed test protocols for PPE with a one-size-fits-all concept. Building on best practice in reviewing the literature and current design requirements, key design parameters were identified such as a minimum strap width and comfort level for healthcare related Face Shield. Two strap headbands made from fabric and elastomer with linear stiffness of 44.1 ± 0.3 N/m and 149.1 ± 3.1 N/m respectively were tested with respect to fit and comfort on small and large arc-shaped models. There was an exponential change in pressure from the side to the middle of the strap headbands. The high stiffness of the elastomer in a radial set-up influenced the pressure exerted on a wearer’s head when the elastomer strap was used. Meanwhile the coefficient of friction between the fabric strap and arc-shaped model influenced the pressure exerted when a fabric strap was used. The ergonomics of the designed Face Shields supported the one-size-fits-all concept, whereby various gender and head circumferences were considered. The findings in this paper will promote new standards in the design of PPE with a one-size-fits-all target.

## Graphical abstract

**Figure:**
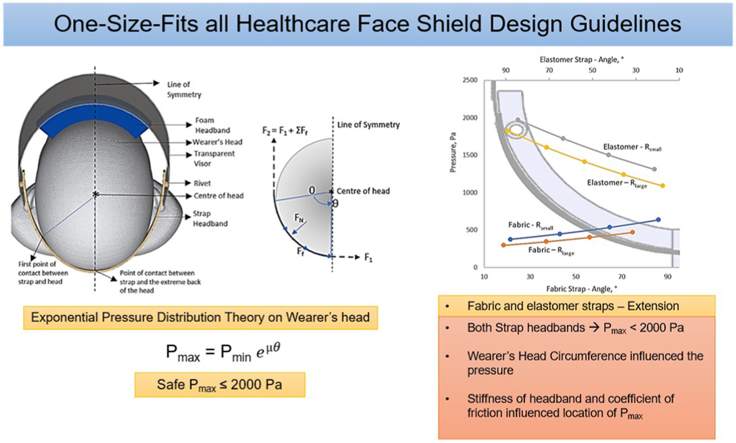

## 1 Introduction

The COVID-19 pandemic required wearers such as healthcare staff, all over the world, to use personal protective equipment, PPE, to prevent them from either catching the infection or transmitting it to others. With the aim of managing the crisis in England, 32 billion of PPE items were procured by the NHS between February to July 2020 [1]. Due to the high international demand, there was a challenge in the supply chain of PPEs worldwide, one of the factors being China's decline in exports [1].

Traditionally, NHS England's procurement depends on the Department of Health and Social Care (DHSC) which is mainly responsible for the NHS budget, NHS England (independent of the DHSC) and Public Health England which oversees the PPE stock during the pandemic [2]. The three organisations were prepared to respond to a worst-case influenza pandemic [3]. However, the COVID-19 pandemic exceeded the expected PPE demand which instigated the Secretary of State for Health and Social Care to bring in the armed forces to support the PPE supply chain [3]. Temporarily, individual NHS Trusts were also purchasing their own PPEs from local small suppliers [4], given that a large number of doctors could not have access to gowns and eye protection [5].

One such PPE, used to alleviate the risk of cross contamination of the SARS-CoV-2 virus for individuals working in close proximity to potentially infected individuals, is the Face Shield. The Face Shield is intended to protect the user's face and orifices against contact and splashes of contaminated fluid and needs to be worn with a filtering face piece class 3 (FFP3) respirator (BS EN166-2002; EU PPE Directive 425). A one-size-fits-all single-use Face Shield is intended to be inexpensive at the point of purchase, straightforward to put on from an individually wrapped clean package and can be safely discarded after one use only. Nonetheless its design and fabrication need to follow the required regulations such as EU PPE Directive 425 and related standards such as BS EN166-2002- Personal Eye Protection.

**Fig. 1:**
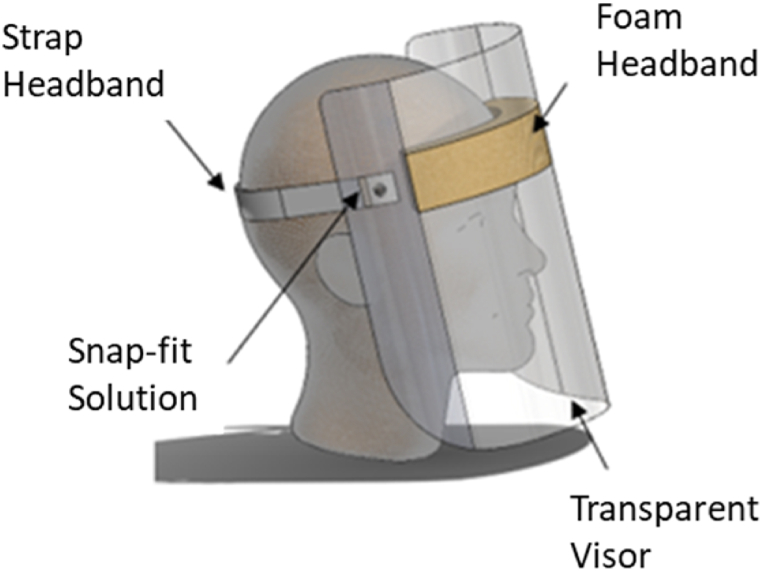
One-size-fits-all single-use Face Shield with the different parts identified.

Fig. 1 shows the basic components of a one-size-fits-all single use Face Shield being foam front head band, strap headband, snap-fit solution, and a transparent visor.

Facial anthropometric dimensions vary significantly between people of different genders, racial/ethnic group, and age range [6]. Derbyshire Healthcare has a majority of 80% staff registered as female and more than 50% of staff being above the age of 45 years [7]. The University Hospitals of Derby and Burton (UHDB) NHS Foundation Trust estimate 21% of their staff to be of black and minority ethnic group [8]. Consequently, a one-size-fits-all concept would aim to accommodate Face Shield Wearers of those various head dimensions. Although the standards for PPE manufacture were relaxed during the COVID-19 pandemic to accelerate the process of PPE testing [9], there was a need to make sure that PPE were tested to provide protection to front-line staff. One of the key requirements from the regulations were for the face shield to be comfortable for the wearer over a long shift (8 Hours in the NHS). Thus, enhancement of the functional design of a Face Shield was also dependent on ergonomic factors [10].

There is a lack of information on the range of flexibility for a one-size-fits-all PPE such as a Face Shield. There is no evidence that a one-size-fits-all PPE can provide the same amount of protection to users of different gender, age, and racial/ethnic group. In a crisis such as the COVID-19 pandemic, there was no guarantee that individuals of all genders, age and ethnic group or individuals wearing accessories such as glasses, turbans and head coverings were equally protected when using one-size-fits-all Face Shield. There is a need to identify gaps in current regulatory standards and assess the fit and function of current self-adjustable PPE with the aim of enhancing design guidelines for such PPE.

In collaboration with the local NHS Trust (University Hospitals of Derby and Burton (UHDB) NHS Foundation Trust and a local small and medium enterprise (SME), Riverside Medical Packaging Ltd, the University of Derby (UoD) aimed at demonstrating enhanced fit and function for a one-size-fits-all Face Shield across different wearers that complies with all regulatory requirements. This will aid in establishing design guidelines to enable a wider range of materials and construction techniques, and therefore reduce the risk of single supplier dependency.

### 1.1 National regulatory standards

In England, the main requirement for a Face Shield to be used in a healthcare setting is the CE marking with respect to BS EN 166:2002, BS EN 167:2002 and BS EN 168:2002 (details in Table 1) [[11], [12], [13]]. PPE manufacturers also follows the PPE Regulation (EU) 2016/425 to be able to sell their product to the European market [14]. Nonetheless there is no specific standards related to the design of Face Shields to be used in a healthcare setting [15]. The above-mentioned regulatory standards provide various detailed guidance, mainly, on the design of the transparent visor. The only detailed requirement for the headband can be found in BS EN 166:2002 - ‘*Headbands, when used as the principal means of retention, shall be at least* 10mm *wide over any portion which may come into contact with the wearer's head. Headbands shall be adjustable or self-adjusting.’* Although following this requirement will allow a Face Shield to be CE marked, such type of headband have been shown to result in PPE associated headache when used for more than 4 h [16]. One cause of such type of headache is the reduction of oxygen and increase in carbon dioxide level in the blood stream due to long hours of wearing face mask and Face Shield [17]. Another cause is the external compression due to the headband, resulting in a continuous stimulation of the cutaneous nerves of the head [17,18]. Similar compression headache has been associated with helmet wearing as well, prevailing mainly on the forehead [19,20].

**Table 1:** Regulations around PPE with headbands.

Regulations	Information related to Face Shield Design
BS EN 166:2002 Personal eye protection. Specifications	Mandatory CE marking requirement. Design, Quality of materials, Increased robustness, stability at high temperatures; Headband width >10 mm and should be adjustable.
BS EN 167:2002 Personal eye protection. Optical test Methods	Optical tests
BS EN 168:2002 Personal eye protection. Non-optical test methods	Requirements for strength, durability, and resistance to impact, Lateral protection
PPE Regulation (EU) 2016/425	Provides protection, Light, no side effect, Sufficient ventilation, prevent moisture, no restriction to vision, compatible with other wearable accessories.
PPE Regulation (EU) 2016/425; Recommendation for Use	Rapid testing of eye- and face protectors against SARS-CoV-2
BS EN 13819-1: 2020 Hearing protectors. Testing. Physical Test methods.	Earmuff and headband adjustment – cushion pressure ≤4500 Pa or force ≤14 N
BS ISO 18527-3:2020 Eye and face protection for sports use. Requirements and test methods for eyewear intended to be used for surface swimming.	Strength: 40 N has sufficient extension capability; Test require extension to 190 ± 10 mm. Slip resistance: 10,0 ± 0,1 N load applied for 60 cycles within 600 ± 10 s.

Similar headbands used for swimming eyewear have been considered in BS ISO 18527-3:2020, as shown in Table 1. The main consideration to the headband was the strength and slip resistance that aligns closely with the design for a Face Shield. However, this standard concentrates on the performance in the presence of water. Another standard, BS EN 13819-1:2020, provides information for headband for hearing protectors whereby the main consideration is the cushion pressure over the ear within a certain range so that the right hearing protection is provided. All the mentioned regulatory standards highlight that the headband must be comfortable for the wearer. Gerges et al. (2010) developed Contact Pressure or Force Distribution Index (SAM1) to quantify the comfort level of ear protectors [21]. The SAM1 index relates to the force distribution per unit area (pressure) on the ear with the optimum comfort present when this pressure is uniform over all contact surface [21]. Weight of the wearable equipment has also been associated with comfort whereby ≤245 g was acceptable for ear protectors [22].

None of the above-mentioned requirements and measuring indices, considers the effect of the pressure on the wearer's head. On a forehead, the venous pressure ranges typically between 400 and 650 Pa while the capillary pressure is about 2700 Pa [23]. For headbands with sensors to measure the blood oxygen level from the forehead, a headband pressure between 500 and 2700 Pa is favoured with more preference to those pressures closer to 2700 Pa to obtain the best reading [23]. Similar observations were made by Agashe et al. (2006) when the headbands' pressure on healthy adults were found to be between 1200 and 2000 Pa [24]. The researchers concluded that the applied pressure below 2000 Pa provided the best oxygen saturation reading and hence did not affect the capillaries or veins adversely. The pressure measurements in both works mentioned above was taken at a single point, with the assumption of the pressure distribution being uniform.

In this paper, a theory was developed to show that pressure distribution on a wearer's forehead is non-uniform and exponential, when similar PPE with elastic headband is worn. The theory was then tested using 2 types of strap headbands.

### 2 Pressure distribution theory

Circumferential stress defines the force distribution per unit area between strap headband and the wearer's head, if there was a uniform pressure exerted by the headband on the wearer's head [25]. However, in this work, it was hypothesised that the pressure distribution is not uniform along the length of the headband in contact with the wearer's head.

**Fig. 2:**
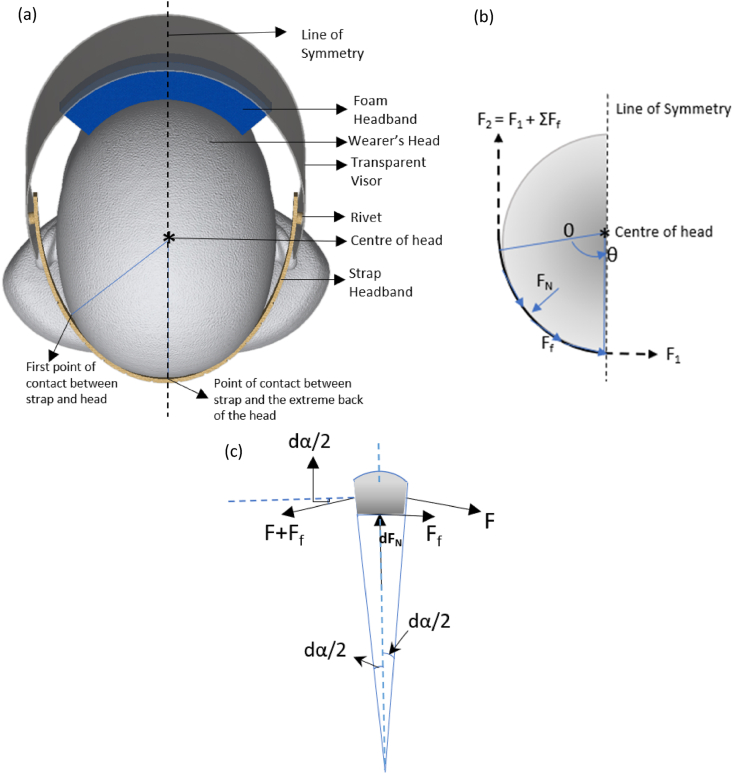
(a) Top view of Face Shield on a wearer's head highlighting the centre of the head, line of symmetry and contact between strap headband and the head, (b) Free body diagram of the different forces acting on half of the strap headband: at the points of contact with the head, normal to the strap headband and due to friction when considering half head and (c) a differential portion of the strap headband to help define the frictional and normal force.

Fig. 2 illustrates the force distribution on the strap headband when in contact with the wearer's head. Fig. 2(a) shows the top of Face Shield as worn by a wearer highlighting the contact area of the strap headband and the head. Only half of the head and Face Shield was considered as a symmetrical force and pressure distribution was assumed. A free body diagram describes the different forces acting on the strap headband due to the tension (F_1_), friction (F_f_) and reaction (F_N_) as shown in Fig. 2(b), assuming a circular head cross section. The direction of the frictional force between the strap and the wearer's head depends on the direction F_1_. The angle, θ, defines the 2 extreme points of contact between strap headband and the head (for half symmetry), as depicted in Fig. 2(a) and (b).

Fig. 2(c) focuses on one differential element of strap headband when in contact with the head. The tension in the strap headband is now defined as F and the normal force as dF_N_.

When considering the half symmetry of the head, the imbalance between F_1_ and F_2_ will determine the direction of the frictional force. [If friction is acting in the opposite direction, to what is shown in Fig. 2(b), then the location of F_1_ and F_2_ is reversed. Coulomb's law of friction can hence be applied [26] to relate the frictional force to the normal force as follows:

(1) $${\text{dF}}_{f}=\mu {\text{dF}}_{N}$$

where μ = coefficient of friction and dF_N_ = normal force.

Assuming dα/2 is small,

(2) $$\frac{{dF}_{f}}{F}=\mu \;d\alpha$$

Integrating (_) as follows,

(3) $$\int_{F1}^{F2}\frac{{dF}_{f}}{F}=\mu \;\int_{0}^{\theta }d\alpha$$

(4) $$F_{2}=F_{1}\;e^{\mu \theta }$$

The differential pressure being exerted on the person's head, dP could thus be defined by the following equation when considering friction:

(5) dP = μ P dα

Integrating equation (5),

(6) $$\int_{P1}^{P2}dP=\mu \int_{0}^{\theta }d\alpha$$

(7) $$P=P\left(0\right)\;e^{\mu \theta }$$

where P is the maximum pressure exerted on the wearer's head (at F_2_) and P (0) is the minimum pressure at the end where F_1_ is acting. If F_1_ happens to be on the first point of contact between the strap headband and the head and F_2_ at the extreme back of the head, then the frictional force (F_f_) will be in the opposite direction. This will result in the minimum pressure P (0) being at the first point of contact between the strap headband and the head and P(θ) at the other end.

In this work, it was hypothesised that a head with a bigger circumference will result in a higher-pressure exertion on the wearer's head. Before the pressure exerted can be calculated, the comfort fit of a Face Shield with respect to the elastic headband need quantifying. As such a fit and function test was first done to relate the range of head circumferences to an applied load.

## 3 Materials and methods

Before any test was performed, the right materials for the Face Shield had to be selected. This process was done in conjunction with the local SME Riverside Medical Packaging Ltd. Only those materials available during the pandemic were selected following which they were tested within the remit of the function of a healthcare Face Shield.

Following material selection, an experimental study was conducted to assess the design and function of a Face Shield using 2 types of adjustable headbands. Subsequently a user evaluation was done using 30 clinical practitioners/trainees above the age of 18 years. The 30 volunteers were provided with instructions and sample Face Shields to put on in their home following which they filled a questionnaire about the fit and comfort of each Face Shield tried on.

### 3.1 Material selection

**Fig. 3:**
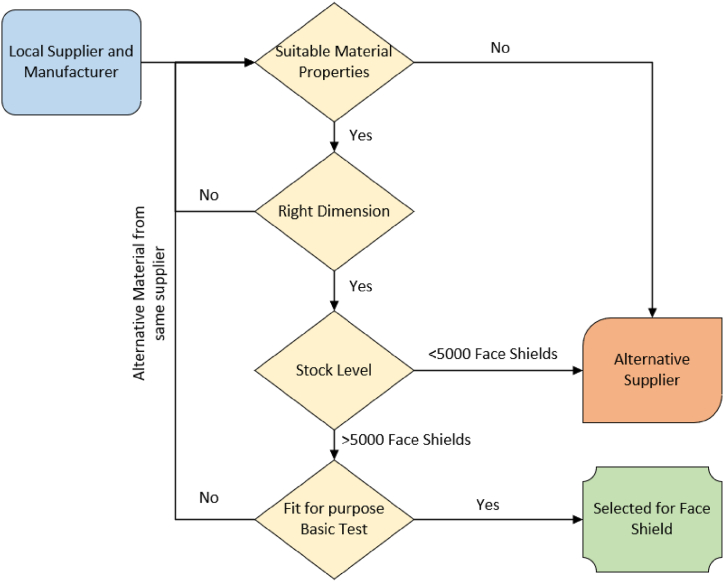
Flowchart identifying the material selection process for the Face Shield.

During the pandemic individual NHS Trusts were allowed to procure various supplies such as PPE locally [4]. In this work, a local supply chain was developed to support the UHDB with their supply of Face Shields. Fig. 3 illustrates the material selection process for the individual parts for the Face Shield manufacturing.

Riverside Medical Packaging Ltd supported the material selection process with their input on the cost effectiveness of the individual parts. The SME created a new manufacturing line for Face Shield in their factory to aid the process. Table 2 narrows down the suppliers of the individual parts which had enough supply to provide the parts in bulk.

**Table 2:** Local suppliers of the parts of the Face Shield.

Face Shield Part	Supplier name	Location
Foam Headband [PU Foam]	Direct Foam Products Ltd & Derby Gaskets Ltd	Wolverhampton, West Midlands Derby, Derbyshire
Snap-Fit Solution [Nylon Rivet]	JetPress Ltd	Sutton in Ashfield, Nottinghamshire
Flexible Strap [Fabric Strap headband] [Elastomer Strap headband]	H Seal & Co Ltd Timesco Healthcare Ltd	Coalville, Leicestershire Basildon, Essex
Transparent Visor [APET Sheet]	DuPont Teijin Films	Dumfries, South Scotland

Briefly, the foam headband, as displayed in Fig. 1, was a polyurethane (PU) foam with an 80% of open cells with a density of 25.2 kg/m^3^ (BS EN ISO 845:2009) and hardness ranging between 110 and 150 N (BS EN ISO 2439: Method B 2008) [27]. The snap fit fastening solution was a light duty nylon rivet with a head diameter of 12.7 mm and a work thickness of 3.2 mm. The APET sheets for the transparent visor had thicknesses of 175 and 250 μm to vary the weight of the Face Shields (For user evaluation). Both strap headbands were elastomeric. However, one of them were woven with a fabric, hence labelled as the fabric strap and the other one was made of an elastomer only and hence labelled elastomer. The fabric strap headband was 20 mm wide, and the elastomer strap was 25 mm wide.

### 3.2 Face shield design

The elementary design of the Face Shield is illustrated in Fig. 1, with the individual parts identified. This paper concentrates on that part of the design that, primarily, affects the one-size fits-all concept, the flexible strap. Firstly, the individual straps were assessed when worn by a medium-head person in a Fit and Function Test. The resulting output were quantified and used to characterise the behaviour of the straps and the resulting pressure on a wearer's head. This was done by a linear and radial strap elongation test.

#### 3.2.1 Fit and function test

The fit and function test, as per its name, ensured the fit of the flexible strap for a comfort fitting of a Face Shield on a wearer's head as identified in the various regulations for PPE in Table 1.

The Face Shields were fabricated as shown in Fig. 1, using the parts in Table 2. The APET sheets were 199 by 325 mm. The Foam Headband had a dimension of 230 by 28 by 25 mm and was self-adhesive. It was adhered to the middle top of the APET sheet. The individual flexible strap was held to the sides of the APET sheets using 1 pair of nylon rivet on each side. The length of the flexible strap was then optimised.

A person with a medium head size (Radius, R = 91 mm) put on the Face Shield with the 2 types of flexible strap, the fabric, and the elastomer band separately. The lengths of the respective strap headbands were varied from 275 mm up to the length that was perceived as comfortable for the wearer. The increment in length for this task was 5 mm for each try-out resulting in the 280, 285, 290 mm and so on. The comfort length for the fabric and elastomer band was concluded to be 295 mm and 310 mm respectively.

Using the optimised comfort length for each strap headband, the Face Shields were worn by the medium head size wearer (n = 3 for each type of strap headband). The average extension for a comfort fitting, experienced by the individual flexible strap, were 80 ± 0.5 mm for the fabric band and 25 ± 0.5 mm for the elastomer band.

#### 3.2.2 Linear strap elongation test

**Fig. 4:**
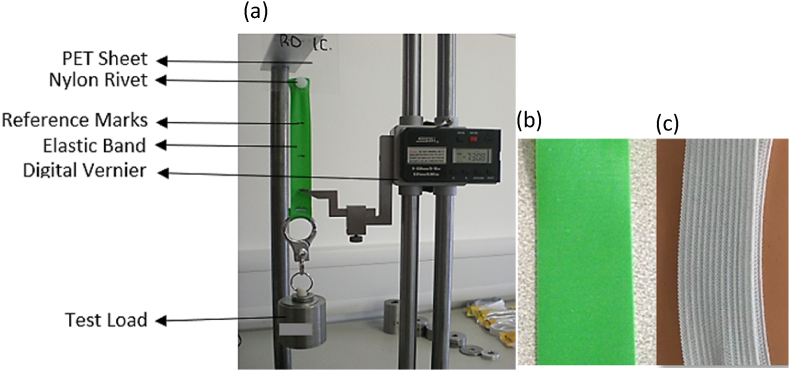
(a) The Experimental set up for linear strap elongation test. Sample section of the (b) elastomer and (c) fabric headband.

A linear strap extension test was developed, as shown in Fig. 4(a), to quantify the load versus extension characteristic of the 2 strap materials. A close view of the elastomer and fabric headband is shown in Fig. 4(b) and (c) respectively. A piece of the individual strap (295 mm for the fabric and 310 mm for the elastomer band) was attached to a section of APET sheet (80 by 150 mm) by a pair of nylon rivet. The APET sheet was held by a clamp on top. Reference marks were sketched on the strap to identify 120 mm in the middle of the latter to normalise the elongation measurements. A digital vernier was used to measure the extension.

Firstly, a test load was attached at the end of the strap with the help of a carabiner. The load was changed until the extension for a comfort fitting, as per the fit and function test, was obtained, that is, 80 ± 0.5 mm for the fabric band and 25 ± 0.5 mm for the elastomer band. This process was repeated 3 times to obtain an average load value. The extension between the reference marks (initial length: 120 mm) was recorded as well. When the load was removed, the strap length was measured again to ensure that each strap returns to its original length. This prevented plastic deformation.

Using the average load value and the reference extension, the linear stiffness of the individual strap material was calculated using the following equation:

(8) $$K=\frac{F}{\delta }$$

F is applied test load in N and δ is the extension in m.

Using the linear stiffness value, the stiffness for a radial set up was calculated. It was assumed that the full length of each type of strap headband is made of 2 springs in series for the test in Fig. 4. However, when applied on a person's head, the springs will be in parallel instead of being in series. Hence the radial stiffness of the strap headband can be calculated as follows:

(9) $$K_{R}=4K$$

#### 3.2.2 Radial strap elongation test

Once the strap materials were characterised linearly, their stiffness were characterised radially. This will reflect their performance when worn on a head as part of a Face Shield. A radial strap elongation test as depicted in Fig. 4, was developed to do so.

Two arc shaped models, with radii 86 and 102 mm to represent a small and large head respectively, were 3 d printed using an Ultimaker 2+ at default setting for Polylactic acid, PLA. The small model was labelled R_small_ and the large one R_large._ A sample strap headband of both material of the comfort length as defined in the fit and function test was attached to a rectangular piece of APET sheet (80 by 150 mm) by a pair of nylon rivet on each side. Eleven markers were placed on the strap headband with 30 mm distance between each of them. The distance between the last marker on each side and the respective rivet was 10 and 12.5 mm for the fabric and elastomer bands respectively. The distance between markers and/or the rivets were used measure the extension of each section separately.

**Fig. 5:**
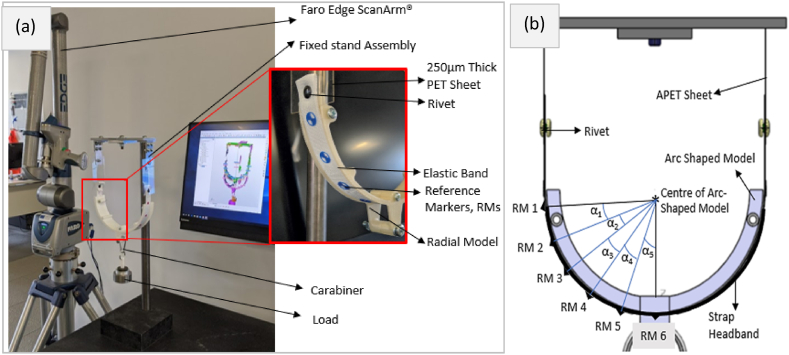
(a) Radial strap elongation test set-up and (b) simplified radial set up identifying the various angles with respect to the reference markers.

The other end of the APET sheet was fixed to the top of the test stand as shown in Fig. 5(a). First the R_small_ model was placed on the fabric band. The resulting length of the different sections was considered as original length. With the help of a carabiner, a load of 0.1, 0.2, 0.3, 0.4 and 0.5 kg was applied to the fabric band separately. The resulting change in length of the 10 sections of the strap headband was digitally recorded using a FARO Edge ScanArm® with an accuracy of ±0.041 mm. This experiment was repeated 5 times. Then the R_large_ model was applied on samples of the woven strap headband to record the extension for the 5 different load applications in 5 repeats. The same was repeated for R_small_ and R_large_ models applied to the elastomer band samples separately, with similar data collected. For both head models, it was ensured that the strap not in contact with the model was vertical.

As mentioned before, the force and pressure distribution on the head/model by the strap headband was assumed to be symmetric and hence only half of the measurements will be considered. Since 5 repeats were done, the average of the 5 elongation tests were considered for additional calculation. Applying equations (9) and (4), the varying forces from α_1_ to α_4_, as shown in Fig. 5(b) was calculated followed by the coefficient of friction between the strap headband and model. Subsequently the maximum pressure exerted by the strap headband on the model was calculated using the total angle between the first and the middle reference marker (Fig. 4(b): α_1_ + α_2_ + α_3_ + α_4_). The maximum pressure value, the individual angle values and the coefficient of friction value were then used to evaluate the pressure distribution from α_1_ to α_4_.

#### 3.2.1 Pressure calculation

Assuming a symmetry in force and pressure distribution on a wearer's head only the measurements from only half the strap headband (first marker to middle of strap headband) was considered in this section. The extension between the rivet and the first marker was not included in the pressure calculation as the individual headband in that section was not in contact with the head model. The 2 forces identified in Fig. 2(b) were calculated as follows:

(10) $$F_{2}=\frac{Applied\;Load}{2}\text{;}$$

F_1_ = k_R_ × δ; (Eq. (1))

where k_R_ is the stiffness of the strap headband in a radial set up and δ is the resulting extension.

(11) $$F_{1}=k\times \left(R\theta \;–\;l_{0}\right)$$

Where R is the radius of the head, θ is the angle between the middle of the strap headband and the first marker when on the model and l_0_ is the initial length of the strap headband that resulted in that specific extension. The friction coefficient was then calculated by applying equation (4). In this scenario, the maximum pressure can be calculated by considering the normal force using the following equation:

(12) $$P_{\max}=\frac{dF_{N}}{R\theta W}$$

Where W is the width of the strap headband. Equation (7) could then be used to calculate P (0). It is to be noted, when the maximum force is at the middle of the template, the associated pressure will be maximum at the middle. And if the maximum force is close to the first marker on the side, then the maximum pressure will be towards the side.

### 3.3 Ergonomics – user evaluation

**Fig. 6:**
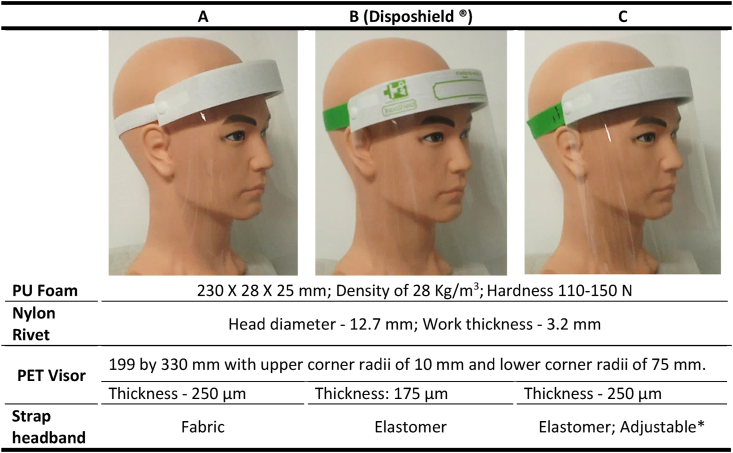
The 3 sample Face Shields presented to the NHS (A, B and C) with the specification of the individual parts used. (* There were 3 markings (S (Small), M (Medium) and L (Large)). The rivet would be initially placed at M. Then if it is too tight or too loose the wearer could remove it and place it accordingly. Instructions were provided along with the Face Shield.).

Poor ergonomics around the fit of PPE have been identified in various sectors previously [28]. During the COVID-19 pandemic there has been a resistance to wearing PPE such as the Face Shield which has been associated with personal beliefs about its inefficacy [29]. In this work, a user evaluation was conducted to understand healthcare users’ experiences on using 3 different types of Face Shields as shown in Fig. 6 which highlight the 2 variants being the headband and the PET visor.

Following the approval of the University of Derby Research Ethics Committee, 30 NHS staff and UoD mature student nurses above the age of 18 years were recruited. They received the 3 types of Face shields each with the instructions on how to put on and off and the list of tasks to do when wearing it for a minimum of 20 min (sit down, stand up, raise arms/shoulders, look up, look down, quick head movements, walk quickly, close-up vision test, long-distance vision test). The tasks would be performed at home and not in their workplace. Subsequently they would need to answer a questionnaire of 10 questions covering the comfort, ease of use and impact on their activities (Refer to supplementary information).

**Fig. 7:**
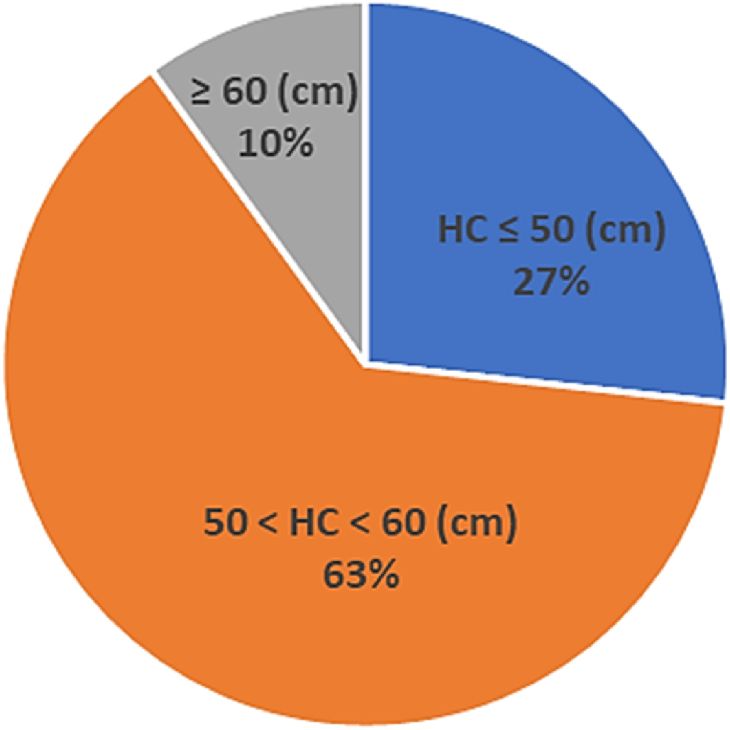
Distribution of head circumferences of Face Shield users for user evaluation.

There were 22 people who identified themselves as female and 8 as male who were recruited. Among those recruits, 75% wore glasses. Their head circumferences varied in size as shown in Fig. 7.

#### 3.3.1 Statistical analysis

The collected data from each multiple-choice question were first averaged. A Kruskal Wallis test, a non-parametric procedure, was then performed for differences between the individual answers for the 3 types of Face Shield. If there was a significant difference between the answers for the 3 groups (n = 90, p > 0.05), a Mann-Whitney test was performed to distinguish the differences between the individual samples. Both tests were performed at 95% confidence interval.

## 4 Results and discussion

The fit and function test confirmed the functional and comfort length of the individual strap headbands to be used as part of a Face Shield, following which their individual linear and radial stiffness was obtained. The radial stiffness values led to the quantification of the pressure distribution on a wearer's head when used with the output of the radial elongation test.

### 4.1 Strap headband testing

The loads required to meet the target extension for the fabric and the elastomer were 0.36 kg and 0.38 kg respectively. The linear stiffness was calculated to be 44.1 ± 0.3 N/m and 149.1 ± 3.1 N/m for the fabric and elastomer headband respectively. This resulted in the radial stiffness to be 176.4 N/m and 596.4 N/m for the fabric and elastomer headband correspondingly.

**Fig. 8:**
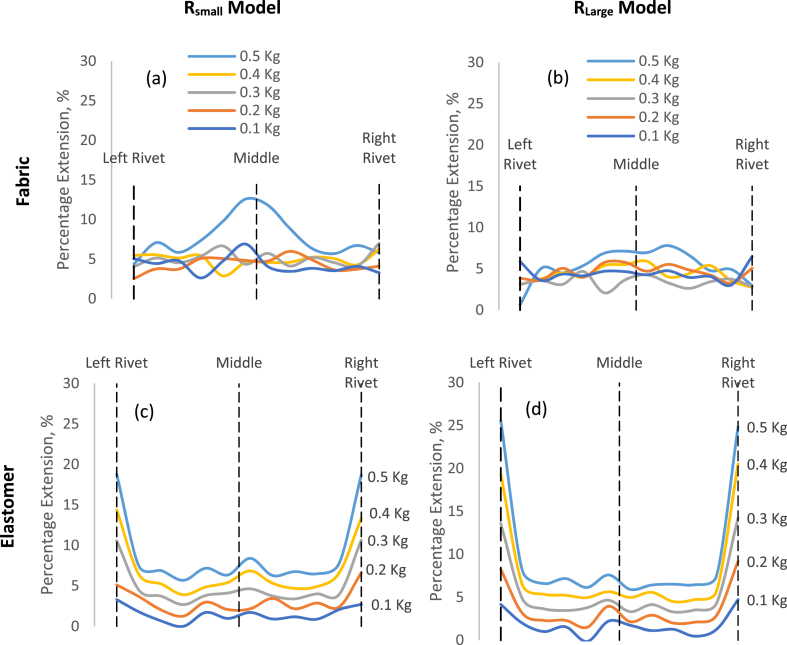
Percentage extension of the (a) fabric strap in the presence of the R_small_ model, (b) fabric strap with the R_Large_ model, (c) elastomer with R_small_ model and (d) elastomer with the R_Large_ model.

During the radial elongation test, for both types of strap headbands, the sum of the individual extensions increased linearly with an increase in the applied load on both R_small_ and R_large_ models, with a symmetric extension distribution. Fig. 8 illustrates the percentage extension for each section of the 2 strap headbands in the presence of the 2 head models. For the fabric strap, the percentage extension along the whole length of the strap ranged between 0 and 5% when a load of 0.1, 0.2, 0.3 and 0.4 kg were applied to both models, R_small_ and R_large._ Furthermore, there was no difference in the percentage extension when the fabric strap was in contact with the models (between rivets) or not (between rivet and first reference marker) (Fig. 8(a) and (b) respectively). This suggested that between 0.1 and 0.4 Kg loading the friction was minimum between the fabric strap and models which allowed less than 5% extension along the whole length of the strap headband. At 0.5 Kg loading on the R_small_ model, there was a 12.6% extension in the middle section of the fabric strap. In the presence of the R_large_ model, the 0.5 Kg loading resulted in a 7% extension in the middle section of the fabric strap. The higher extension in the middle of the fabric band as compared to the side for 0.5 Kg could be better explained by the effect of frictional force. Using Eq. (4), the coefficient of friction, μ, for the contact between the fabric strap and the R_small_ and R_large_ models were 0.47 and 0.49 respectively with the direction of friction being towards the middle of the models. A lower coefficient of friction for the R_small_ model resulted in a higher normal force, F_N_. To react to the higher normal force, the fabric strap experienced a greater extension in the presence of the R_small_ model as compared to the R_Large_ model.

Fig. 8(c) and (d) represent the extension of the elastomer headband. The extension between the first and last marker (when in contact with the arc model), the elastomer strap exhibited similar extension behaviour along the strap headband in the presence of 0.1–0.5 kg, as compared to the fabric strap. The extension at both ends (Between the rivet and the first marker), which were not in contact with the models, was higher at all loads and model size as compared to those sections in contact with the models. The stiffness of the elastomer strap was about 4 times higher than that of the fabric ones. This resulted in a significantly lower extension of the elastomer when in contact with the R_small_ and R_large_ models causing the elastomer to extend more when not in contact with the models, to accommodate the applied loads. The distribution of the extension in the elastomer in contact with the models was uniform and within the similar percentage extension range as for the fabric ones (<5%) for each load. However, when the load increased from 0.1 to 0.5 kg, there was a uniform increase in the percentage extension of the elastomer strap. This was also associated with the higher stiffness of the elastomer which resisted extension in the presence of loads.

Using equation (4), the coefficient of friction, μ, for the contact between the elastomer strap and the R_small_ and R_large_ models were 0.37 and 0.41 respectively with the direction of friction being away from the middle of the models. This difference did not significantly affect the extension of the elastomer when in contact with the R_small_ and R_large_ models.

For a Face Shield with the Fabric strap, the frictional force between the wearer's head and the strap headband will determine the behaviour of the strap with respect to the wearer's head size. Nevertheless, when for a Face Shield with an elastomer strap, it will be the stiffness value that will influence the behaviour of the material more.

### 4.2 Pressure distribution

From the fit and function test, a load of 0.36 and 0.38 Kg were found to represent a comfortable fit for the fabric and elastomer strap headband. To represent the mentioned loads, the extension data from Fig. 8 and load 0.4 Kg was used for the pressure calculation on a small and large head along the length of the strap headband. For each angle, α, the pressure being applied on the models was calculated and presented in Fig. 8. Both type of strap headbands exhibited a maximum pressure of less than the previously recommended 2000Pa and hence were concluded to be safe to wear over an 8-h shift. Nonetheless the maximum pressure exerted by the elastomer was close to 2000 Pa and hence is borderline safe.

**Fig. 9:**
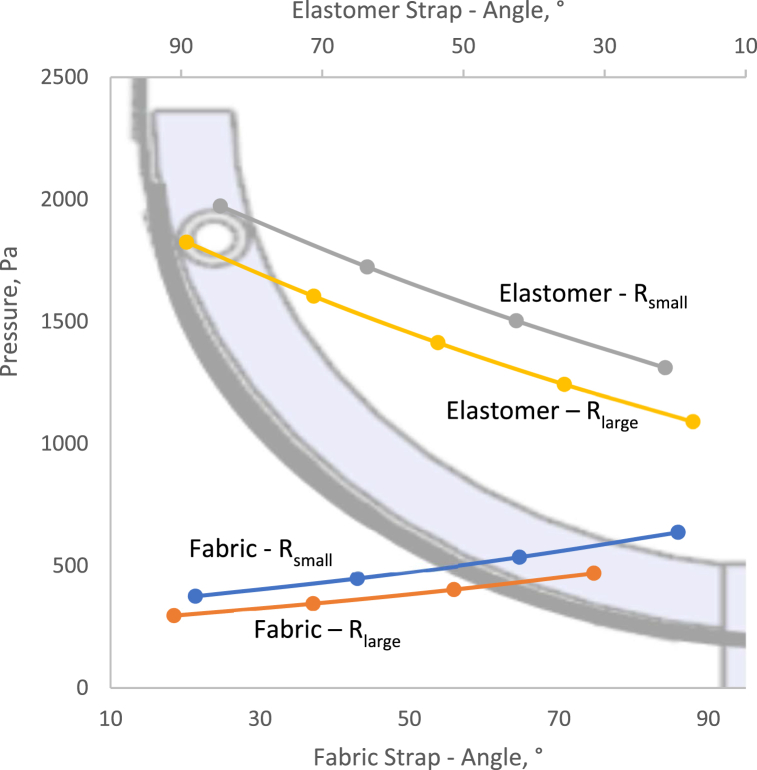
Pressure calculated between each reference markers because of the applied load, model circumference and strap headband material.

From Fig. 9, it was observed that the maximum pressure exerted on the template due to the elastomer (1972 Pa) was higher than that exerted by the fabric (638 Pa). The elastomer had a higher stiffness and hence a higher tension experienced by the strap headband in the presence of a load. This accounted for the higher pressure exerted on the models by the elastomer.

For both types of strap headband, the pressure exerted on the R_large_ model was lower than that on the R_small_ model. The coefficient of friction in the presence of either strap headband was significantly lower in the presence of the R_small_ model as compared to that of the R_large_ model. The higher friction coefficient resulted in more resistance to move when in contact with the template. Consequently, once a headband is in contact with the R_large_ model or head, the friction between them both prevents movement more as compared to when in contact with the R_small_ model. Thus, more extension occurs in the strap headband in contact with the template as compared to the contact-free section (between rivet and first marker). This is confirmed by Fig. 8 where the extension between the rivet and first marker is lower in the presence of the R_small_ model as compared to the R_large_ model) with a higher percentage extension throughout the strap headband in contact with the model.

Another difference was that the maximum pressure was on the side of the model for the elastomer and minimum at the middle for both R_small_ and R_Large_ models. The opposite was observed for the fabric strap, with the minimum at the side end and maximum at the middle. This was also associated with the higher stiffness of the elastomer as compared to the fabric. Fig. 8 emphasized on the significantly higher percentage extension of the elastomer section when not in contact with the model. This will result in a higher pull force on the elastomer sections in contact with the model, towards the rivet. This was reflected by the higher force acting on the side of the elastomer resulting in P(θ) acting on the side. For the fabric strap headband, although the extension was not significantly higher in the middle section of the strap, there was a biased increase in the percentage extension of the fabric strap towards the middle. This accounted for higher tension in the middle section of the strap headband for the same load resulting in P(θ) being in the middle of the headband.

When a person is wearing a Face Shield, the fit will also be dependent on the front frame and in the case of the single use Face shield, the PU foam. To confirm the impact of the strap headband on the pressure exerted on a wearer's head, the effect of the foam used could be done. Future work could involve analysis of the compression, density, and hardness of the foam in combination with the properties of the individual strap headbands.

### 4.3 Ergonomics – user evaluation

All 3 types of Face Shields were found to be easy to put on, adjust to comfort and take off. All the users were able to perform the requested tasks without any identified issues. Nonetheless, all of them acknowledged the bottom edges of the Face Shield touching their body during use. Thus, the Face Shield touching their body was not considered as something hindering their tasks.

The fit of Face Shield B was concluded to be significantly better that A and C from the user evaluation (Kruskal Wallis, p = 0.026; Mann-Whitney, p = 0.01 (A and C)). The difference between A and B was associated with the higher stiffness of the elastomer (449.6 N/m) as compared to the fabric (106.2 N/m) when part of the Face Shield's radial set up. A higher stiffness indicates a higher ability to resist deformation or motion of the strap headband, hence maintaining immobility and fit on the wearer's head. The difference between B and C was related to the higher coefficient of friction of the elastomer when used at the fixed initial length of 305 cm (∼0.4). In the case of C, when the wearer adjusts the strap headband according to their perceived comfort level, the coefficient of friction will change with respect to the head size and change applied. Thus, the adjustable function was found not to add any value to the Face Shield when being used among wearers with head dimension ranging between 53 and 63 cm.

Equal number of users experienced fogging during use as compared to those who did not. As per BS EN 166:2002, a Face Shield must be able to resist fogging for a minimum of 8 s of exposure [11]. Further investigation is required to be able to confirm the ability to resist fogging. If this test is not passed, then possible use of antifogging coating/film, change to design of transparent visor and/or use of alternative material to PET should be considered.

### 5.0 Conclusions, limitations, and future research

The University of Derby collaborated with the University Hospital Derby and Burton NHS Trust and a local SME, Riverside Medical Packaging Ltd to develop Face Shield Designs to accommodate the front-line staffs in the hospital. The focus was on the one-size-fits-all concept with respect to the fit and comfort.

Following a fit and function test, 2 different strap headbands (Fabric and Elastomer) were tested with respect to the fit and comfort of a wearer and the factor of safety for similar PPE. Subsequently, 3 Face Shield demonstrators were manufactured with those straps to be evaluated by healthcare staffs. The following conclusions were made:

1. The elastomer had a significantly higher radial stiffness (596.4 N/m) than the fabric strap (176.4 N/m). Hence, the comfort length for the fabric and elastomer, for a Face Shield, were 295 and 310 mm respectively requiring a 27% and 8% extension during fitting correspondingly.
2. The coefficient of friction, predominantly, influenced the extension of the fabric strap when in contact with the arc-shaped models. However, it was the stiffness which mainly influenced the extension of the elastomer strap when in contact with the arc-shaped models, irrespective of the size of the model.
3. The R_small_ model experienced higher pressure than the R_large_ model when in contact with the strap headbands irrespective of the applied load and type of strap headband. And the maximum pressure when using both types of strap headbands were below 2000 Pa which makes them safe to be used for 8-h shifts.
4. The direction of the force of friction was towards the middle of the arc-shaped models for the elastomer and the contrary was observed for the fabric strap. These observations were associated with the higher stiffness and coefficient of friction respectively.
5. The user evaluation reinforced the experimental data with significant differences with respect to fit and comfort.

This study was limited by the number of variables determining the length of the headband, the sample size for user evaluation and duration of donning a Face shield. Future studies could investigate the effect of the foam headband on the pressure exerted on larger samples of wearers with varying head morphology for a longer period.

## Author contribution statement

Urvashi F Gunputh: Conceived and designed the experiments; Analyzed and interpreted the data; Wrote the paper.

Gavin Williams, Adam Leighton: Performed the experiments.

Wayne Carter: Performed the experiments; Analyzed and interpreted the data.

Hirbod Varasteh, Melinda Lyons: Analyzed and interpreted the data.

Marzena Pawlik, Yiling Lu, Jenny Clementson, Gyan Tripathi, Nick Chambers: Analyzed and interpreted the data; Wrote the paper.

Ruth Sim: Conceived and designed the experiments; Analyzed and interpreted the data.

Matt Roe: Contributed reagents, materials, analysis tools or data.

Paul Wood: Conceived and designed the experiments; Wrote the paper.

## Funding

This paper was partly funded by the 10.13039/100008961College of Science and Engineering of the 10.13039/100010025University of Derby. The materials and manpower for face shield manufacture and assembly was funded by Riverside Medical Packaging Ltd.

## Data availability statement

Data will be made available on request.

## Declaration of competing interest

The authors declare that they have no known competing financial interests or personal relationships that could have appeared to influence the work reported in this paper.
