# Supplementary material for: Design and manufacture of one-size-fits-all healthcare face shields for the NHS during the COVID-19 pandemic
Source: Heliyon. 2023 Sep 4;9(9):e19368. doi: 10.1016/j.heliyon.2023.e19368 (PMC10558341; doi:10.1016/j.heliyon.2023.e19368)
Supplement: Multimedia component 1 [file mmc1.docx]

**Procedure and questions**

Thank you for agreeing to take part in this study, it is very much appreciated.

You will have several face visors. These have different size visors and we are interested in how they fit, how comfortable they are, etc. Please answer all questions honestly and openly – there are no wrong answers!

(**Link to Questions:** <https://derby.qualtrics.com/jfe/form/SV_2lUeMWekPaZQxwi>)

**Please choose one of the visors. Make a note of the code on the side of the visor here:**

Please put on the face visor – adjust the headband until the visor feels secure.

Please wear the visor for 20 minutes. Please complete the following tasks whilst wearing the visor:

*Crouching down (as if to get something out of a low cupboard)*

*Extend neck to look forward (as if to look at something on the far side of a patient in a bed)*

*Turn your head to both left and right*

*Sit down / stand up*

*Raise your shoulders / arms*

*Look up at the ceiling*

*Look down at the floor*

*Move your head quickly up and down/side to side*

*Walk quickly*

*Test your close up vision - Looking at the fine detail of an object close-up (e.g. as if threading a needle)*

*Test your long-distance vision (see if you can read a number plate at 20 metres)*

*Test visual comfort while looking at an object (e.g. watching tv / looking at a computer screen)*

*Test whether you can be easily be heard by others / can easily hear.*

Please note you should only attempt the stated tasks if that is something you would normally do and are happy to do so, but not to attempt any movements that you would ordinarily avoid or are likely to cause you discomfort – just skip that/those tasks.

There will be questions to complete in relation each face visor after wearing them which can be found online at <https://derby.qualtrics.com/jfe/form/SV_2lUeMWekPaZQxwi>

Now please answer these questions – select whichever response you feel best captures your opinion for *this* visor.

How did you find putting the face visor on?

1 very easy

2 quite easy

3 neither easy or difficult

4 quite difficult

5 very difficult

How did you find adjusting the face visor?

1 very easy

2 quite easy

3 neither easy or difficult

4 quite difficult

5 very difficult

How did you find completing tasks whilst wearing the face visor (in terms of any impact the visor had on task completion)?

1 very easy

2 quite easy

3 neither easy or difficult

4 quite difficult

5 very difficult

How did you find adjusting to the visibility through the face visor?

1 very easy

2 quite easy

3 neither easy or difficult

4 quite difficult

5 very difficult

How comfortable was the face visor?

1 very comfortable

2 comfortable

3 neither comfortable or uncomfortable

4 uncomfortable

5 very uncomfortable

How well did the face visor fit?

1 very well

2 quite well

3 neither well or not well

4 not too well

5 not well at all

How easy do you think it would be to remove the face visor without risk of contamination?

1 very easy

2 quite easy

3 neither easy or difficult

4 quite difficult

5 very difficult

Did you have any issues with fogging of the face visor?

Yes / No

Did the face visor touch your shoulder(s), chest or other part of your upper body during the tasks you completed?

Yes / No

If you’d like to give any details about any of the above answers please do so here

Repeat for each visor

Please make sure the questions are answered online so that they can be analysed. (<https://derby.qualtrics.com/jfe/form/SV_e8rrG4at13I2VoN>)
